# Genotype-phenotype relationship in hereditary amyotrophic lateral sclerosis

**DOI:** 10.1186/s40035-015-0036-y

**Published:** 2015-07-24

**Authors:** Satoshi Yamashita, Yukio Ando

**Affiliations:** Department of Neurology, Graduate School of Medical Sciences, Kumamoto University, 1-1-1 Honjo, Kumamoto, 860-8556 Japan

**Keywords:** Amyotrophic lateral sclerosis (ALS), Genotype, Phenotype, Sporadic ALS (SALS), Familial ALS (FALS)

## Abstract

Amyotrophic lateral sclerosis (ALS) is the most common adult-onset motor neuron disease. It is characterized by neuronal loss and degeneration of the upper motor neurons (UMNs) and lower motor neurons (LMNs), and is usually fatal due to respiratory failure within 3–5 years of onset. Although approximately 5–10 % of patients with ALS have an inherited form of the disease, the distinction between hereditary and apparently sporadic ALS (SALS) seems to be artificial. Thus, genetic factors play a role in all types of ALS, to a greater or lesser extent. During the decade of upheaval, the evolution of molecular genetics technology has rapidly advanced our genetic knowledge about the causes of ALS, and the relationship between the genetic subtypes and clinical phenotype. In this review, we will focus on the possible genotype-phenotype correlation in hereditary ALS. Uncovering the identity of the genetic factors in ALS will not only improve the accuracy of ALS diagnosis, but may also provide new approaches for preventing and treating the disease.

**Electronic supplementary material:**

The online version of this article (doi:10.1186/s40035-015-0036-y) contains supplementary material, which is available to authorized users.

## Introduction

Amyotrophic lateral sclerosis (ALS) is the most common adult-onset motor neuron disease. It is characterized by progressive neuronal loss and degeneration of the upper motor neurons (UMNs) and lower motor neurons (LMNs). The demise of motor neurons causes the central nervous system (CNS) to lose the ability to control voluntary muscle movement, eventually resulting in death due to respiratory failure in the later stages of the disease.

The cause of ALS remains an enigma. However, approximately 5–10 % of patients with ALS have an inherited form of the disease. During the decade of upheaval, the evolution of molecular genetics technology has rapidly advanced our knowledge about the genetic causes of ALS. Familial ALS (FALS) has been attributed to mutations in at least 24 different genes. Some mutations in FALS-related genes have been identified in patients with sporadic ALS (SALS). Because the initial symptoms of ALS vary across patients, a diagnosis of ALS can be established by excluding various diseases mimicking ALS. Smooth and reliable diagnosis is the first step in the good clinical management of patients with ALS. Therefore, genetic testing might be a helpful tool for diagnosing FALS as well as SALS with mutations in FALS-related genes.

It is important, but difficult, to predict which genes are most likely to be implicated in some patients with ALS. A diagnostic algorithm could improve the accuracy of a genetic explanation. Therefore, we review the possible genotype-phenotype relationship in ALS cases with mutations in the FALS-related genes. Uncovering the identity of the genetic factors in ALS will not only improve the accuracy of ALS diagnosis, but may also provide new approaches for preventing and treating the disorder.

## Classification of hereditary ALS

Hereditary ALS can be transmitted as a dominant, recessive, or X-linked trait, but the most common type is an adult-onset disorder with autosomal dominant transmission. Autosomal recessive inheritance is rarer and frequently seen in patients with juvenile onset ALS, primary lateral sclerosis (PLS), or spastic paraplegia-like symptoms. X-linked dominantly inherited ALS is a rarely-observed condition, seen in families where male patients tend to show more severe phenotypes. We demonstrate the characteristic phenotypes in each type of FALS, and summarize them in Table 1.

**Table 1:** The genotype and phenotype associated with familial ALS-related genes

Type	Gene	Mode of inheritance	Country	Age at onset (range)	Mean age at onset (years)	Initial symptoms	UMN	Cognitive impairment	Other features
ALS1	SOD1	AD, AR, de novo	Japan, Italy, Spain, Korea, UK, USA, Turkey, Sweden, Iran, Porland, Bulgaria, China, France, Germany, Denmark, Pakistan, Canada, and so on	6–94	48	LL > UL > bulbar	Positive (LMN dominant)	Very rare	Progressive muscular atrophy, progressive bulbar palsy, facial onset sensory motor neuronopathy (FOSMN) syndrome, vocal cord paralysis, cerebellar ataxia, sensory disturbance (vibration), autonomic dysfunction (incontinence, neurogenic bladder), lower back pain
ALS2	Alsin	AR	Tunisia, Saudi Arabia, Kuwait, Italy, Algeria, Hungary, Germany, The Netherlands, Pakistan, Bangladesh, Turkey, Japan, Portugal, France, Cyprus, China	1–11	2	LL, UL	Positive	None	Juvenile ALS, juvenile primary lateral sclerosis, infantile-onset ascending hereditary spastic paraplegia, generalized dystonia, cerebellar ataxia
ALS3	unknown	AD							
ALS4	SETX	AD	USA, Austria, Belgium, Italy, Afghanistan, China	1–73	19	LL > UL	Positive	None	Cerebellar ataxia, oculomotor apraxia (type 2), motor neuropathy, thin cervical spinal cord
ALS5	SPG11	AR	Italy, Turkey, Japan, Canada, Brazil	7–23	16	Bulbar, LL, UL	Positive	Rare (mental retardation)	Juvenile ALS, hereditary spastic paraparesis, autonomic dysfunction (incontinence)
ALS6	FUS	AD, AR, de novo	Belgium, Italy, Korea, UK, Japan, Turkey, Canada, France, USA, Germany	13–80	45	UL, bulbar > LL	Positive (LMN dominant)	Rare (mental retardation)	Progressive muscular atrophy, Parkinsonism, essential tremor, schizofrenia, learning disabilities
ALS7	unknown	AD							
ALS8	VAPB	AD	Brazil, UK, France (Japan), The Netherlands	18–73	44	UL, LL	Negative	None	Progressive muscular atrophy, progressive bulbar palsy, motor neuropathy, postural tremor, autonomic dysfunction (chronic intestinal constipation, sexual dysfunction)
ALS9	ANG	AD	The Neitherland, Ireland, Scotland, UK, USA, Sweden, Italy, France, Germany, China,	21–86	55	UL, LL, bulbar	Positive	FTD	Parkinsonism, progressive bulbar palsy
ALS10	TDP-43	AD, AR	Italy, France, UK, China, Germany, Turkey, USA, Belgium, Japan, Porland, Afghaistan, Canada	20–77	54	UL, LL, bulbar	Positive	FTD (rare)	Parkinsonism, chorea, progressive supranuclear palsy
ALS11	FIG4	AD	USA	29–77	55	Bulbar > UL, LL	Positive	None	Hereditary spastic paraparesis, primary lateral sclerosis, personality change
ALS12	OPTN	AD, AR	Japan, Italy, Turkey, The Netherlands, Denmark	24–83	51	Bulbar, UL, LL	Positive	FTD, AGD	Primary open angle glaucoma, parkinsonism, finger deformity, personality change, depression
ALS13	ATXN2	AD	USA, Belgium, the Netherlands, Canada, France, China, Germany, Switzerland, Italy, Turkey, Cuba	21–87	60	UL, LL	Positive	None	Cerebellar ataxia, corticobasal syndrome, Parkinsonism
ALS14	VCP	AD	Italy, USA, The Netherlands, Japan	36–68	48	LL > UL > bulbar	Positive	FTD	Paget’s Disease, inclusion body myopathy
ALS15	UBQLN2	SD	USA, Australia, Canada, Italy, Turkey, Belgium, Germany, Bulgaria	M: 14-72, F: 16-77	44	UL, LL, bulbar	Positive	FTD	Primary lateral sclerosis, progressive bulbar palsy, relentlessly progressive choreoathetoid movements, spastic paralysis
ALS16	SIGMAR1	AD	Saudi Arabia	1-68	1	LL > UL	Positive	FTD (rare)	Juvenile ALS
ALS17	CHMP2B	AD	Denmark, the Netherlands	26-73	69	Bulbar, UL, LL, respiratory	Positive (LMN dominant)	FTD	Progressive muscular atrophy, parkinsonism
ALS18	PFN1	AD	Sephardic Jewish, Italy, USA, China, Belgium	33-63	53	Limb	N/A	N/A	
ALS19	ERBB4	AD	Japan, Canada	45-70	61	UL, bulbar, respiration	Positive	None	
ALS20	HNRNPA1	AD	N/A	N/A	N/A	N/A	N/A	FTD	Paget’s Disease, inclusion body myopathy
ALS21	MATR3	AD	USA,UK, Italy, Taiwan	36-64	52	LL > UL, bulbar	Positive	FTD	Distal myopathy (inclusion body myopathy)
ALS-FTD1	C9ORF72	AD	Finland, Sardinia, Ireland, UK, Italy, USA,Canada, Germany, the Netherlands, Turkey, Israel, Australia, Japan	27–80	57	UL, LL, bulbar	Positive	FTD	Parkinsonism, cerebellar ataxia, psychosis,
ALS-FTD2	CHCHD10	AD	France, USA, Germany, Spain, Italy, Finland	35-73	56	Bulbar, UL, LL	Positive (LMN dominant)	FTD	Cerebellar ataxia, mitochondrial myopathy, deafness, neurogenic bladder, facial paresis, Parkinsonism
	TBK1	AD, de novo	Sweden, Denmark, Germany, France, Portugal	35-80	60	Bulbar, UL, LL, respiratory	Positive	FTD (50 %)	

*AD*, autosomal dominant; *AR*, autosomal recessive; *UL*, upper limb; *LL*, lower limb; *LMN*, lower motor neuron; *FTD*, frontotemporal dementia

### ALS1: Cu/Zn superoxide dismutase 1, soluble (SOD1)

**Fig. 1:**
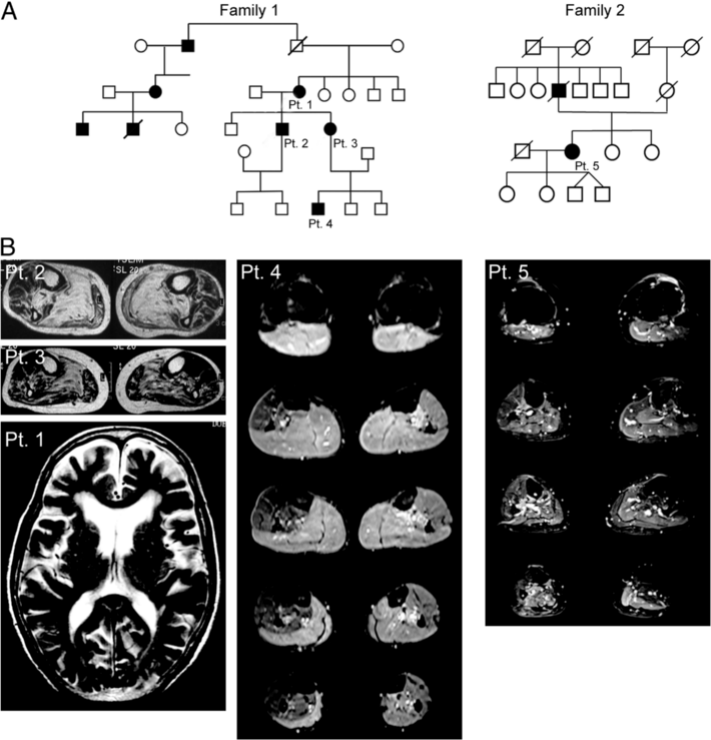
Two FALS families with the SOD1 H46R mutations. **a** Family trees of Family 1 and 2. All of the patients showed the same uniform feature that initial symptoms were restricted to the flexor muscle group in the unilateral distal leg. **b** Short-T1 inversion recovery MR images revealed high intensity lesions in the gastrocnemial and soleus muscles of the patient 2, 3, 4, and 5. Brain MRI of patient 1 showed frontal lobe-dominant atrophy

In 1991, Siddique et al. [1] showed the linkage of FALS to chromosome 21q by positional cloning and demonstrated genetic locus heterogeneity in FALS. Rosen et al. [2] then reported a genetic linkage between FALS and a gene encoding cytosolic Cu/Zn superoxide dismutase (SOD1)—a homodimeric metalloenzyme that catalyzes the reaction of toxic superoxide anion O_2_^−^ to O_2_ and H_2_O_2_. Since SOD1 missense mutations were established as the first causative genes for ALS, the number of known mutations has increased to more than 185 so far (Additional file 1: Table S1). Most cases were inherited in an autosomal dominant manner, but the D90A mutation transmitted the disease in both an autosomal dominant and autosomal recessive manner. Globally, the most frequent SOD1 gene mutation is D90A. However, in the USA, the most frequent mutation was A4V, and in the UK and Japan, the most common mutations were I113T, and H46R, respectively. However, to our knowledge, no SOD1 mutation was reported from Ireland. Regarding clinical features of ALS with the SOD1 mutation, lower limb-onset and predominant LMN involvement are relatively common (Table 1). The D90A-homozygous mutation is associated with slowly progressive paresis in the legs that gradually spreads up to the arms, thoracic and bulbar musculature, with atypical non-motor features such as ataxia, neuralgic, aching pain, heat sensations, and bladder disturbance. Interestingly, it has been reported that patients with SOD1-related FALS greatly differed with respect to the age of onset of weakness, while the duration of the disease appears to be characteristic for each type of mutation. Some SOD1 mutants (D90A-homozygous, E100K, E100G, A89V, L84F, L84V, D76V, H46R, G37R, and G10V) tend to show a uniform phenotype, while other mutants (A4V, C6G, G41S, N86S, D90A-heterozygous, I112M, I113T, L144F, and V148I) have greatly variable phenotypes. The A4V, H43R, L84V, G85R, N86S, and G93A mutations have been associated with rapid disease progression and survival times shorter than 3 years, whereas the cases with G93C, D90A, or H46R mutations exhibit longer life expectancies, up to more than 10 years after disease onset [3–5]. These findings suggest that each type of SOD1 mutation may be associated with a different degree of toxicity. We examined two unrelated FALS families with H46R mutations (Fig. 1). The patients showed a uniform phenotype: the initial symptom was unilateral weakness of the flexor muscles in the distal lower limbs (Fig. 1) [6]. This might be attributed to mitochondrial respiratory chain dysfunction due to mutant SOD1 expression in the muscles as previously reported [7].

### ALS2: Alsin

To date, more than 50 patients with mutations in the Alsin gene have been reported with early onset of the disease (~1 year). These patients generally belong to Middle Eastern, European, and Mediterranean countries, Japan, and China (Additional file 1: Table S1). All patients with ALS2 had homozygous or compound heterozygous mutations in the Alsin gene. Mutations in the Alsin gene cause three distinct disorders: infantile ascending hereditary spastic paraplegia (IAHSP), juvenile primary lateral sclerosis (JPLS), and autosomal recessive juvenile amyotrophic lateral sclerosis (JALS) (Table 1) [8, 9]. A recent study reported patients with ALS2 with nonsense and frameshift mutations in the Alsin gene who presented with generalized dystonia and cerebellar signs [10]. Although the phenotype-genotype correlation remains undetermined so far, most of the mutations predict truncated proteins, which could be unstable in structure and lose their function.

### ALS4: Senataxin (SETX)

Senataxin (SETX) was initially identified as a causative gene for severe early-onset ataxia with oculomotor apraxia (AOA2), which is the second most common recessive ataxia after Freidreich's ataxia [11]. Later, heterozygous mutations were found in patients with the autosomal dominant form of juvenile-onset ALS [12]. ALS4 is characterized by slowly progressive distal muscle weakness and atrophy with pyramidal signs, sparing of bulbar and respiratory muscles, and frontal dysfunction (Table 1) [13]. So far, the T3I, L389S, T1118I, C1554G, K2018E, K2029E, R2136H, and I2547T mutations in the SETX gene have been identified in both patients with FALS and those with SALS with widely differing symptoms (Additional file 1: Table S1). In a recent report, a patient with late onset ALS4, bulbar involvement, and predominantly proximal distribution of amyotrophy presented with choreic movements and elevated alpha-fetoprotein levels [14]. In contrast, one study demonstrated that previously published ALS4-related missense mutations are most likely to be non-pathogenic and just polymorphisms [15]. Therefore, we should carefully interpret the significance of SETX missense mutations in the absence of functional assays.

### ALS5: Spastic paraplegia 11, autosomal recessive (SPG11)

Mutations in the Spatacsin (SPG11) gene represent the most common form of autosomal recessive hereditary spastic paraplegia with thin corpus callosum (HSP-TCC) [16]. Recently, SPG11 mutations have been identified in patients with the autosomal recessive form of juvenile ALS, indicating a wide clinical spectrum for SPG11 mutations [17]. The SPG11 mutations can be associated with an intrafamilial phenotypic heterogeneity, including atypical ALS and classic HSP-TCC [18]. To our knowledge, at least 28 patients with ALS5 have been described with juvenile onset of the disease, ranging from 7 to 23 years (Table 1 and Additional file 1: Table S1). All patients with ALS5 were associated with slow progression of symptoms with apparent UMN involvement (Table 1). It has been reported that the absence of thin corpus callosum, white matter alterations, cognitive deficits or mental problems clearly differentiates ALS5 from HSP-TCC [17]. At this point, it is unclear why the SPG11 mutations lead to clinical phenotypes resembling ALS or HSP-TCC.

### ALS6: Fused in sarcoma/translocated in liposarcoma (FUS/TLS)

Two independent studies have reported that mutations in the fused in sarcoma/translocated in liposarcoma (FUS/TLS) gene were responsible for ~3 % of FALS and <1 % of SALS cases [19, 20]. FUS/TLS mutations, as well as TAR DNA-binding protein (TDP-43) mutations, have been increasingly reported from Asian countries [21, 22]. Some FUS/TLS gene mutations have been observed in patients with the juvenile form of ALS beginning at younger than 25 years [23–25, 22]. Case studies with the R521C mutation in the FUS/TLS gene emphasized the phenotypes of weakness of the neck and proximal muscles, which may be a clinical hallmark of ALS [26]. Most of the reported cases with the FUS/TLS mutation had no cognitive change. However, some of the patients with juvenile ALS with truncating FUS/TLS mutations have had mental retardation [27, 22].

Most ALS-related FUS/TLS mutations are located at the highly conserved regions of exon 15 that include the non-canonical nuclear localization signal (PY-NLS). Recent studies have shown that the mutations that nullify the PY-NLS lead to redistribution of FUS/TLS to the cytoplasm, where it is recruited into stress granules [28–30]. Notably, the degree of cytosolic mislocalization has been shown to be inversely correlated with the age of disease onset [29]. It has been reported that the truncating mutation R495X was associated with an aggressive disease course, whereas the K510R mutation showed a mild phenotype with disease duration ranging from 6 to 8 years [31].

### ALS8: Vesicle-associated membrane protein-associated protein B (VAPB)

A mutation in the vesicle-associated membrane protein-associated protein B (VAPB) gene was initially reported in Brazilian families with motor neuron disease with a wide range of phenotypes: late-onset spinal muscular atrophy, atypical ALS, or typical ALS [32]. In addition, several patients showed autonomic abnormalities, including chronic intestinal constipation, and sexual dysfunction [33]. So far, the T46I, P56S, and V234I mutations in the VAPB gene have been described in patients from Brazil, Japan, the United Kingdom, and the Netherlands (Additional file 1: Table S1). Further investigation will be required to understand the phenotype-genotype correlation.

### ALS9: Angiogenin (ANG)

A cohort study in Ireland has identified several mutations in the angiogenin (ANG) gene in patients with ALS of Irish and Scottish background, both in familial and sporadic cases [34]. Subsequent clinical studies confirmed the association of these mutations with ALS, and identified new mutations in people with backgrounds from Brazil, China, France, Germany, Italy, Netherlands, Sweden, and the USA (Supplementary Table 1). Frontotemporal dementia (FTD) was also reported in a large FALS pedigree with the K17I ANG mutation [35]. Moreover, a relationship between mutations in the ANG gene and Parkinson’s disease has been revealed [36].

### ALS10: TAR DNA-binding protein (TDP-43)

Several groups have identified mutations in a highly conserved region of TDP-43 in SALS and FALS cases [37–40]. Most mutations are located in exon 6, which encodes the conservative glycine-rich domain. The phenotype and genotype analysis study in patients with ALS having TDP-43 gene mutations revealed that they had earlier onset (53.4 years; range 28-78), predominantly upper limb onset (60.7 %), and longer disease duration (63.0 months; range 32.0-77.2), compared with those having SALS [41]. In Caucasians, 51.3 % of the patients had the upper limb onset, whereas 58.8 % of Asian patients had bulbar onset [41].

### ALS11: FIG4 homolog, SAC1 lipid phosphatase domain containing (S. cerevisiae) (FIG4)

Mutations in the FIG4 gene are responsible for the recessive form of Charcot-Marie-Tooth disease (CMT4J), with early onset and involvement of both sensory and motor neurons [42]. Subsequently, the same group identified ALS as a rare manifestation of the gene [43]. The phenotype observed in patients with FIG4 mutations is still controversial. Some patients carried a diagnosis of definite or probable ALS, and other patients were diagnosed with PLS, associated with predominant UMN involvement. Personality changes were also reported in patients with ALS11.

### ALS12: Optineurin (OPTN)

Maruyama et al. [44] identified mutations in the optineurin (OPTN) gene in 3.8 % of Japanese with FALS and 0.29 % of Japanese with SALS. Mutations in the OPTN gene were also detected in some patients with both FALS and SALS in cohorts of Italian, Danish, French, Turkish, and German patients (Additional file 1: Table S1). As mentioned later, the role of OPTN in the pathogenesis of ALS has been further examined in a recent publication on the TANK-binding kinase (TBK1) gene [45, 46]. The clinical phenotypes of OPTN-related ALS showed relatively slow progression and long duration before respiratory dysfunction, but the onset age of the eight individuals with mutations of OPTN ranged from 30 to 60 years [44]. Brain atrophy with personality change or depression was also observed in patients with ALS12.

### ALS13: ataxin 2 (ATXN2)

Long polyglutamine tracts, including more than 34 CAG repeats in the ataxin 2 (ATXN2) gene, have been identified as a cause of spinocerebellar ataxia type 2 (SCA2) [47]. Recent studies revealed that intermediate-length polyglutamine repeats (between 24 and 33) within the ATXN2 gene can be a risk factor for patients with ALS in different ethnic groups [48–50]. However, whether the clinical features of patients with ALS can be affected by ATXN2 intermediate-length repeats is still controversial [49–51].

### ALS14: Valosin-containing protein (VCP)

Using exome sequencing, Johnson et al. [52] identified a R191Q mutation in the valosin-containing protein (VCP) gene in an Italian family with autosomal dominantly inherited ALS. Screening of the VCP gene in a cohort of ALS cases identified several mutations including a pathologically proven case of ALS. Mutations in the VCP gene have previously been identified in families with inclusion body myopathy, Paget disease, and frontotemporal dementia (IBMPFD) [53]. The phenotype of patients with VCP mutations shows intrafamilial variations from IBMPFD to FALS [54]. This suggests that motor neuron disease is part of the clinical spectrum of multiple proteinopathy of VCP-associated disease.

### ALS15: ubiquilin 2 (UBQLN2)

Recent studies have revealed that ubiquilin 2 (UBQLN2), which regulates the degradation of ubiquitinated proteins, plays a pathogenic role in the X-linked form of ALS with or without FTD [55]. In an original case, the disease was transmitted in a dominant fashion with reduced penetrance without male-to-male transmission of the disease. Age at onset was significantly different between male and female patients, with male patients having earlier age of onset [55]. Mutations in UBQLN2 are not a frequent cause of ALS in the Dutch, French-Canadian, French, Irish, Taiwanese, and Korean population (Additional file 1: Table S1).

### ALS16: σNon-opioid receptor (SIGMAR1)

Homozygosity mapping followed by direct sequencing has revealed a mutation in the σNon-opioid receptor (SIGMAR1) gene in patients in a consanguineous family with the autosomal recessive form of juvenile ALS in Saudi Arabia [56]. Furthermore, variants in the 3′-untranslated region (UTR) of the SIGMAR1 gene were reported in patients with frontotemporal lobar degeneration (FTLD) or motor neuron disease with FTLD [57]. However, the same family with the 3′-UTR mutation of the SIGMAR1 gene also had an expansion of a noncoding GGGGCC hexanucleotide repeat in the chromosome 9 open reading frame 72 (C9ORF72) [58]. This indicates that coding and noncoding variants located in the 3'-UTR of the SIGMAR1 gene are not the cause of FTLD-MND.

### ALS17: chromatin modifying protein 2B (CHMP2B)

Mutations in the charged multivesicular body protein 2B (CHMP2B) gene have been initially identified in patients with FTD [59]. Although the phenotype is predominantly FTD, ALS has been reported as a rare manifestation of the gene [60, 61]. Neuropathology of the patient with the mutation showed LMN predominant disease with ubiquitylated inclusions in motor neurons [60]. Thus, classical ALS and PMA without corticospinal findings are phenotypes associated with mutations in the CHMP2B gene.

### ALS18: profilin 1 (PFN1)

Exome sequencing followed by direct sequencing has shown mutations in the profilin 1 (PFN1) gene, which is a central regulator of actin dynamics in some FALS cases [62]. However, cohort analyses of patients with FALS and those with SALS from France and Quebec, Italy, Germany, the Nordic countries, and the United States suggested that the PFN1 mutation is a rare cause of ALS (Additional file 1: Table S1). In the original report, all patients with ALS18 showed limb symptoms at a relatively younger onset [62].

### ALS19: v-erb-b2 avian erythroblastic leukemia viral oncogene homolog 4 (ERBB4)

A whole-genome sequencing and parametric linkage analysis identified the mutation in the v-erb-b2 avian erythroblastic leukemia viral oncogene homolog 4 (ERBB4) gene in patients of a Japanese family with late-onset, autosomal-dominant ALS [63]. An extensive mutational analysis revealed the same mutation in a Canadian individual with familial ALS and a de novo mutation in a Japanese case [63]. As of this moment, the genotype-phenotype correlation has not been determined.

### ALS20: heterogeneous nuclear ribonucleoprotein A1 (hnRNPA1)

Exome sequencing revealed mutations in the heterogeneous nuclear ribonucleoprotein A1 (hnRNPA1) gene in patients presenting with ALS and/or multisystem proteinopathy (MSP). These mutations are associated with a rare and complex phenotype associating FTLD, Paget disease of bone, and inclusion body myopathy [64]. Because the clinical information is not fully available, the phenotype of patients with mutant hnRNPA1 is still unclear.

### ALS21: matrin-3 (MATR3)

**Fig. 2:**
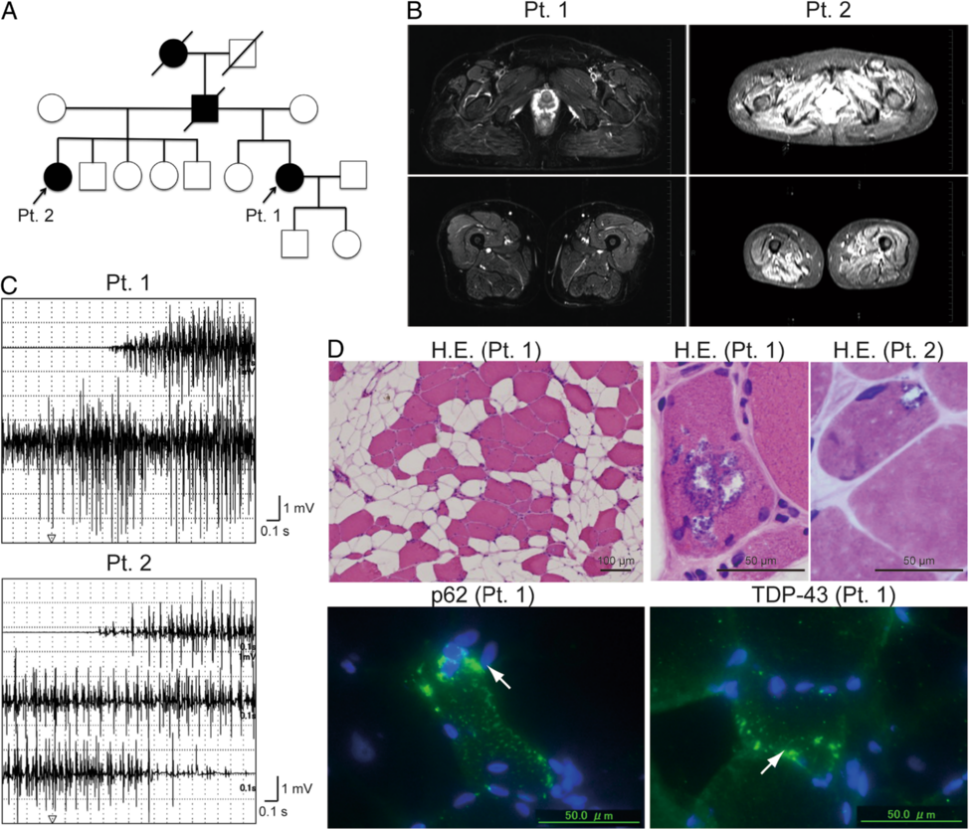
A family with the MATR3 S85C mutation. **a** Family tree of cases with the MATR3 S85C mutation. The detailed clinical information was previously described [67]. **b** Short-T1 inversion recovery MR images revealed fatty and degenerative changes in the gluteus, quadriceps, and hamstring muscles of patient 1 and the paraspinal and gluteus muscles of patient 2. **c** Needle electromyography demonstrated chronic denervation in the vastus lateralis muscles of patients 1 and 2. **d** Muscle biopsy from patients 1 and 2 showed severe fatty and myopathic changes with rimmed vacuoles. Immunohistochemical analysis demonstrated p62- or TDP-43-positive sarcoplasmic granular staining in degenerating myofibers of patient 1. The observation of chronic denervation and renervation on electromyography and muscle biopsy, split hand syndrome, and decremental motor responses to repetitive nerve stimulation (data not shown) suggest the involvement of lower motor neurons in patients 1 and 2

A recent study using exome sequencing revealed mutations in the matrin-3 (MATR3) gene in FALS and FTD cases [65]. Initially, the S85C mutation in the MATR3 gene was reported as the cause of autosomal dominant distal myopathy with vocal cord paralysis (VCPDM) in large multi-generational families [66]. The phenotype observed in some patients carrying MATR3 mutations is still controversial. However, the clinical phenotype might be markedly similar to that observed in patients with mutations in VCP, hnRNPA1, and HNRNPA2B1 as MSP. We examined 2 sisters with VCPDM and S85C mutations in the MATR3 gene (Fig. 2) [67]. Both patients showed no UMN symptoms clinically; however, they showed chronic denervation and renervation on electromyography and muscle biopsy, split hand syndrome, and decremental motor responses to repetitive nerve stimulation, suggesting the involvement of LMNs [67].

### ALS-FTD1: chromosome 9 open reading frame 72 (C9ORF72)

Two independent studies have discovered an expansion of a noncoding GGGGCC hexanucleotide repeat in the C9ORF72 gene that is associated with disease in a large FTD/ALS kindred linked to chromosome 9p [68, 69]. Analysis of extended clinical series found the C9ORF72 repeat expansion to be the most common genetic abnormality in both familial FTD (11.7 %) and familial ALS (23.5 %) [68]. Another study reported that the C9ORF72 intronic expansion was present in 11 % of the cohort, 43 % of FALS cases, and 7 % of SALS cases [69]. Therefore, C9ORF72 has been thought to be the most common cause of ALS in Caucasians, but rarer in other populations [70]. It is still controversial whether the patients with C9ORF72 expansion have shorter disease duration and relatively rapidly progression. C9ORF72 expansion can also cause parkinsonism and dementia. There is no association between the repeat length of the normal alleles, of the repeat in C9ORF72, and disease phenotype or age at onset in C9ORF72 mutation carriers or non carriers [71].

### ALS-FTD2: Coiled-coil-helix-coiled-coil-helix domain containing 10 (CHCHD10)

Whole-exome sequencing identified a missense S59L mutation in the coiled-coil-helix-coiled-coil-helix domain containing 10 (CHCHD10) gene in a large family with a late-onset phenotype including motor neuron disease, cognitive decline resembling FTD, cerebellar ataxia and myopathy [72]. Multiple mitochondrial DNA deletions have been found in the skeletal muscles of patients with ALS-FTD2, suggesting mitochondrial DNA instability. Thus, the phenotype can vary according to affected organs.

### TANK-binding kinase 1 (TBK1)

Recently, several studies using exome sequencing of moderate numbers of patients with ALS identified the TBK1 gene as an ALS gene, which is known to bind to and phosphorylate ALS-related proteins such as OPTN and p62 (SQSTM1/sequestosome) [45, 46]. Patients having ALS with the mutations frequently (~50 %) showed cognitive impairment [46]. Another study performing whole-genome sequencing in patients with FTLD-TDP found variants in the TBK1 gene, indicating a key role for the OPTN/TBK1 pathway in ALS and FTD [73].

## Importance of genetic testing for ALS diagnosis

**Fig. 3:**
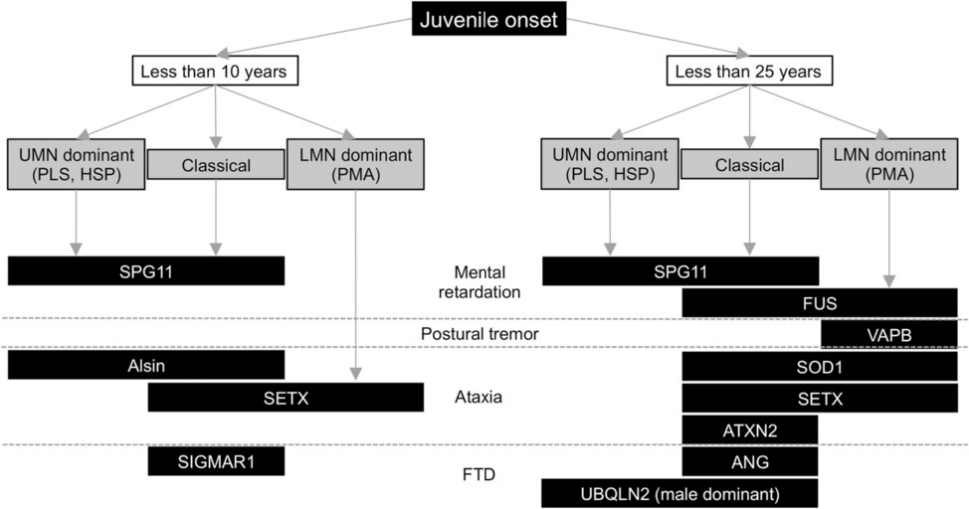
Flowchart for diagnosis of hereditary juvenile-onset ALS

We describe the possible correlation between the genotype and phenotype, and aim to provide a clue to the diagnosis of ALS. ALS cases can be divided into 3 groups: juvenile onset less than 10 years or less 25 years, and adult onset type. Cases with juvenile onset were categorized into 2 groups because we could differentiate the genes that cause juvenile ALS alone from the genes that cause both juvenile and adult-onset ALS. ALS cases with juvenile onset less than 10 years include cases with mutations in the SPG11, Alsin, SETX, and SIGMAR1 genes (Fig. 3). When the symptoms are UMN-dominant, SPG and Alsin can be causative genes for ALS. In contrast, SETX might be responsible in cases with LMN-dominant symptoms such as PMA type. In ALS cases with onset from 10 to 24 years, SPG11, FUS, VAPB, SOD1, SETX, ATXN2, ANG, and UBQLN2 should be considered as a cause of ALS (Fig. 3). SPG or UBQLN2 might be a causative gene in UMN-dominant cases whereas FUS, VAPB, SOD1, and SETX should be examined in LMN-dominant cases.

**Fig. 4:**
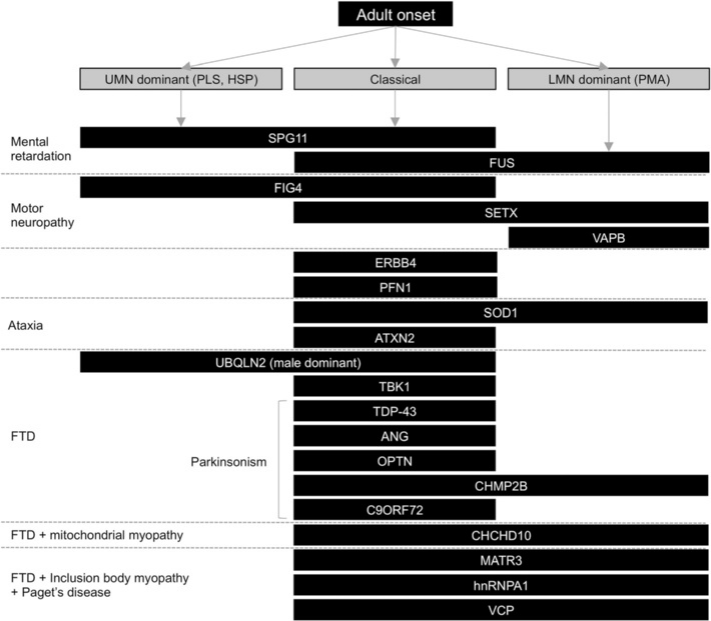
Flowchart for diagnosis of hereditary adult-onset ALS

In adult-onset ALS cases, many candidate genes should be excluded (Fig. 4). In patients who suffer from mental retardation, SPG11 may be responsible in UMN-dominant cases and FUS may be responsible in LMN-dominant cases. Coexistence with cerebellar ataxia may suggest the involvement of mutations of SOD1, ATXN2, Alsin, and SETX. Complications of motor neuropathy might occur in cases with mutations in the FIG4, SETX, VAPB, and SOD1 (homozygous D90A) genes. FTD can be present in cases with mutations in the UBQLN2, SIGMAR1, TDP-43, ANG, OPTN, CHMP2B, and C9ORF72 genes. Moreover, parkinsonism can be involved in cases with TDP-43, ANG, OPTN, and CHMP2B mutations. In some cases, muscle biopsy provides useful information for ALS diagnosis. Mitochondrial myopathy is reported in cases with CHCHD10 and SOD1 mutations (Fig. 1). FTD in combination with inclusion body myopathy and Paget’s disease of bone in the patients or families strongly suggests mutations in the VCP, hnRNPA1, or MATR3 genes (Fig. 2).

Although these algorithms might provide some indications of what type of genetic abnormality might be present in a large enough family with somewhat consistent features, most families have a small number of affected individuals with wide variability. Thus, these algorithms may be ineffective. However, ethnic background plays a huge role in determining which genes are most likely. The proportion of ALS caused by a particular gene in a particular population can be a stronger predictor: C9ORF72 intronic expansion is very common in Caucasians, but rare in other populations. Therefore, the algorithms should be optimized based on ethnic backgrounds, and establishment of panels that examine all genes simultaneously would be ideal.

Another limitation is that phenotypes were described in limited number of patients in some genes except SOD1, FUS/TLS, TDP-43, and C9ORF72. This makes it is difficult to draw conclusive genotype-phenotype correlations. Moreover, some of the reported mutations were not necessarily pathogenic, just polymorphisms [15]. Thus, it is difficult to know which reported variants indeed cause the disease; it even more difficult in cases having oligogenic inheritance because their phenotype is derived from the combination of two genes.

Notably, half of the families with FALS do not have a mutation in the identified genes and therefore the genetic test is not necessarily informative for all cases of FALS. At this point, the determination that an individual has FALS is based on a family history rather than a genetic test. If one's family history is unknown or a parent passed away at a young age, testing is appropriate. Those patients with SALS without a family history can also be offered genetic testing. However, it is extremely important that this be done in the context of genetic counseling or after discussion with a neurologist about the implication of finding a mutation, as a mutation would mean that the ALS is hereditary. Although prenatal genetic testing technology exists, the patients and family members should discuss the procedure with their neurologist and genetic counselor for further information on this complex and personal matter [74].

## Conclusions

There is no specific test or procedure to establish the diagnosis of ALS. A diagnosis of ALS can be established by ruling out other diseases that mimic ALS thorough comprehensive diagnostic examinations. Earlier diagnosis allows prompt initiation with a specific drug, such as riluzole, and accurate palliative care planning. The recent advances in the genetics of ALS have not only contributed to our understanding of the pathogenesis of ALS, but have also provided a tool for diagnostic procedures in some cases of ALS.

Despite all the progress achieved, the large majority of ALS genes remain unknown. The number of genes known to be involved in ALS is expected to continuously increase with the evolution of molecular genetics technology. Further discovery of the genetic factors in ALS will contribute considerably to the diagnosis, care, prevention, and treatment of ALS.

## Additional file

Additional file 1: Table S1. The genotypes and phenotypes in previously-reported ALS cases with familial ALS-related mutations. UMN, upper motor neuron; LMN, lower motor neuron; UL, upper limb; LL, lower limb; FTD, frontotemporal dementia; N/A, not applicable; IAHSP, infantile ascending hereditary spastic paraplegia; JPLS, juvenile primary lateral sclerosis; JALS, juvenile amyotrophic lateral sclerosis; PMA, progressive muscular atrophy. (XLSX 96 kb)

## Abbreviations

ALS: Amyotrophic lateral sclerosis
SALS: Sporadic ALS
FALS: Familial ALS
CNS: Central nervous system
SOD1: Cu/Zn superoxide dismutase
IAHSP: Infantile ascending hereditary spastic paraplegia
JPLS: Juvenile primary lateral sclerosis
JALS: Juvenile amyotrophic lateral sclerosis
SETX: Senataxin
SPG11: Spatacsin
HSP-TCC: Hereditary spastic paraplegia with thin corpus callosum
FUS/TLS: Fused in sarcoma/translocated in liposarcoma
PY-NLS: Non-canonical nuclear localization signal
VAPB: Vesicle-associated membrane protein-associated protein B
ANG: Angiogenin
FTD: Frontotemporal dementia
TDP-43: TAR DNA-binding protein
CMT4J: Charcot-Marie-Tooth disease
OPTN: Optineurin
ATXN2: Ataxin 2
VCP: Valosin-containing protein
IBMPFD: Inclusion body myopathy, Paget disease, and frontotemporal dementia
UBQLN2: Ubiquilin 2
SIGMAR1: σNon-opioid receptor
FTLD: Frontotemporal lobar degeneration
CHMP2B: Charged multivesicular body protein 2B
PFN1: Profilin 1
ERBB4: v-erb-b2 avian erythroblastic leukemia viral oncogene homolog 4
hnRNPA1: Heterogeneous nuclear ribonucleoprotein A1
MSP: Multisystem proteinopathy
MATR3: Matrin-3
VCPDM: Distal myopathy with vocal cord paralysis
C9ORF72: Chromosome 9 open reading frame 72
CHCHD10: Coiled-coil-helix-coiled-coil-helix domain containing 10
TBK1: TANK-binding kinase 1
LMN: Lower motor neuron
UMN: Upper motor neuron
